# FOXC1 restrains NF‐κB‐mediated interleukin‐1β transcription in breast cancer

**DOI:** 10.1002/mco2.440

**Published:** 2023-12-16

**Authors:** Yan Liu, Shuang Chen, Mao Tian, Armando Giuliano, Xiaojiang Cui

**Affiliations:** ^1^ Department of Surgery Cedars‐Sinai Medical Center Los Angeles California USA; ^2^ Department of Biomedical Sciences Cedars‐Sinai Medical Center Los Angeles California USA; ^3^ Jonsson Comprehensive Cancer Center The University of California Los Angeles California USA

Dear Editor,

Basal‐like breast cancer (BLBC) is associated with a poor prognosis and a high mortality rate. Recent studies have established FOXC1 (Forkhead Box C1) as a specific diagnostic and prognostic biomarker for BLBC.^1^ Notably, a number of mRNA profiling studies have further demonstrated that FOXC1 expression correlates with the basal‐like immunosuppressed subtype of BLBC.^2^, ^3^ However, how FOXC1 regulates immune response genes and influences the immunosuppressive tumor microenvironment is poorly understood. Previous studies have shown that FOXC1 can increase NF‐κB activity in breast cancer cells via enhancing p65 protein stability.^4^ The transcription factor NF‐κB plays a key role in regulating immune function and inflammatory responses. Its activity is significantly correlated with BLBC.^5^ It is a critical regulator of interleukin‐1β (IL‐1β), a potent proinflammatory cytokine that can conversely induce NF‐κB activity, thus permitting an autoregulatory feedback loop.^6^ However, IL‐1β is not correlated with BLBC (Figures S1A and B), suggesting that other IL‐1β regulators counteract the NF‐κB‐mediated IL‐1β expression in BLBC. Notably, in a recent study, overexpression of FOXC1 attenuated cigarette smoke‐induced IL‐1β expression as well as oxidative stress, inflammation, and apoptosis in chronic obstructive pulmonary disease,^7^ though the underlying mechanism is unknown.

We hypothesize that FOXC1 may regulate the NF‐κB‐induced expression of IL‐1β. To address this, we stably transfected MDA‐MB‐231 cells with the human *FOXC1* gene (231‐Foxc1 cells). Cells transfected with the empty vector were used as control (231‐Vctrl cells). FOXC1 expression in 231‐Foxc1 and 231‐Vctrl was determined by quantitative real‐time PCR (qRT‐PCR) and immunoblotting (Figures 1A and C). We then treated both cell lines with lipopolysaccharide (LPS), a known inducer of inflammation activating the NF‐κB signaling pathway. As expected, NF‐κB activity was significantly induced in MDA‐MB‐231 cells (Figure S2). Additionally, we determined LPS induced expression of the inflammation‐associated genes IL‐1β, IL‐6, ICAM1, and Cox‐2 in 231‐Vctrl and 231‐Foxc1 cells by qRT‐PCR and immunoblotting (Figures 1B and C). Surprisingly, we found that overexpression of FOXC1 significantly attenuated the LPS‐mediated expression of these genes, with IL‐1β mRNA showing the biggest differences (Figure 1B). Then, we determined IL‐1β, IL‐6, ICAM‐1, and COX‐2 gene expression in both 231‐Vctrl and 231‐Foxc1 cells in response to IL‐1β treatment (5 ng/mL for 6 h). While expression of IL‐1β, IL‐6, ICAM‐1, and COX‐2 mRNA was strongly upregulated by IL‐1β in both 231‐Vctrl and 231‐Foxc1 cells, FOXC1 overexpression significantly diminished IL‐1β‐mediated expression of IL‐1β, IL‐6, and COX‐2, but not ICAM‐1 mRNA, and IL‐1β and Cox‐2 protein expression (Figures S3A and B). These results suggest that FOXC1 may play a critical role in regulating IL‐1β expression and it also affects other inflammation‐associated genes. Therefore, we further investigated the potential regulation of inflammation signal‐elicited IL‐1β expression by FOXC1.

**FIGURE 1:**
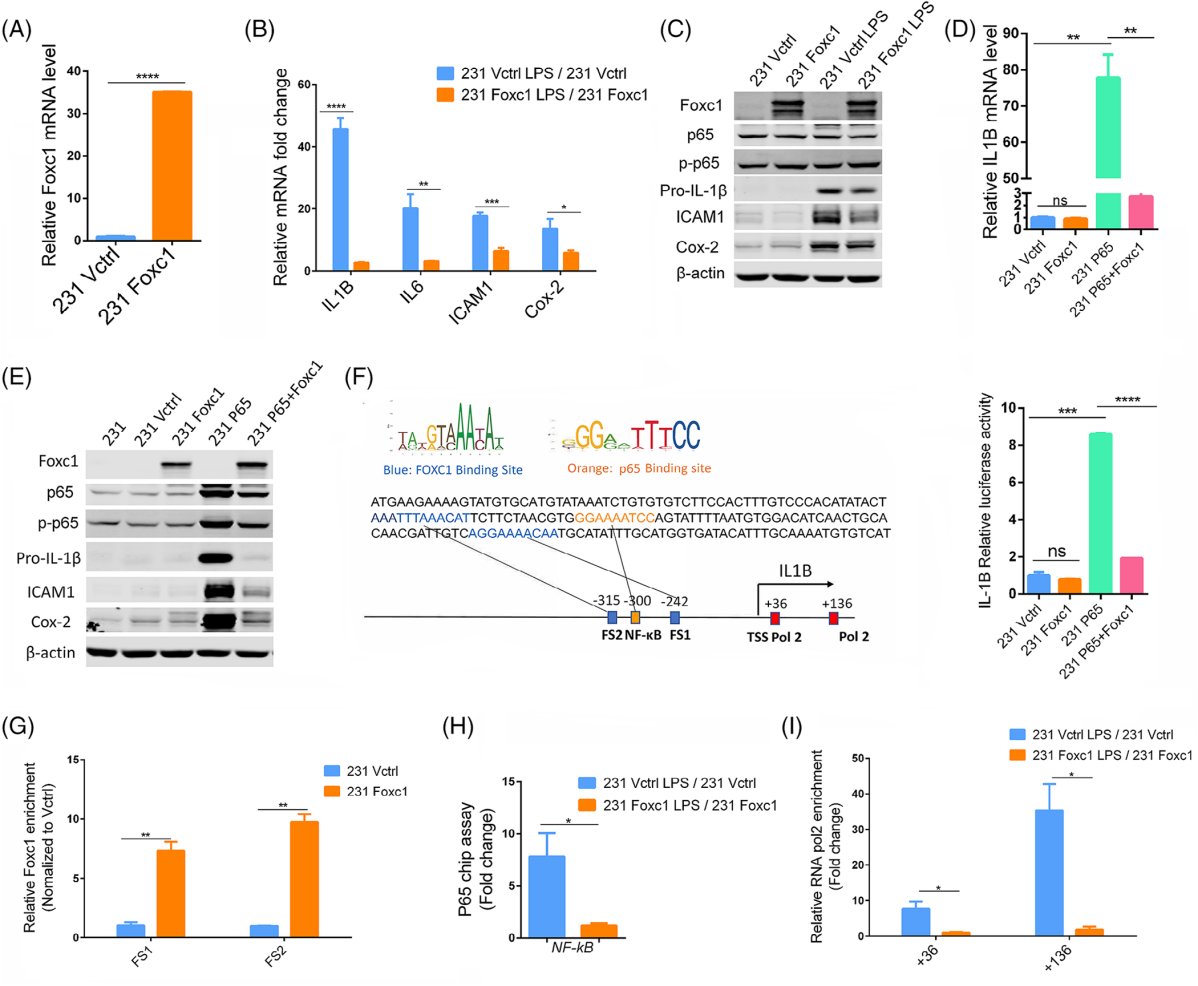
FOXC1 suppresses the p65‐mediated IL‐1β expression by interfering with the interaction of p65 with the IL‐1β promoter. (A) qRT‐PCR analysis of FOXC1 mRNA expression in 231‐Vctrl and 231‐Foxc1 cells (*n* = 3). (B) FOXC1 inhibited inflammatory gene IL‐1β, IL‐6, Cox‐2, and ICAM1 mRNA expression in LPS (1 μg/mL)‐stimulated MDA‐MB‐231 cells. mRNA levels were determined by real‐time PCR. Fold changes of gene expression induced by LPS relative to the vehicle control are presented (*n* = 3). (C) LPS (1 μg/mL) treated 231‐Vctrl and 231‐Foxc1 cells were subjected to western blotting analysis for FOXC1, p65, p‐p65, pro‐IL‐1β, Cox‐2, and ICAM1 protein. (D and E) MDA‐MB‐231 cells transfected with PLNCX2‐control, PLNCX2‐FOXC1, PCMV6‐p65, and co‐transfected PLNCX2‐FOXC1 and PCMV6‐p65 vectors. qRT‐PCR analysis of IL‐1β mRNA expression (*n* = 3) (D); Western blotting analysis of FOXC1, p65, p‐p65, pro‐IL‐1β, Cox‐2, ICAM1, and β‐actin protein expression (E). (F) The left panel illustrates predicted FOXC1‐binding sites (FS1, FS2) and NF‐κβ binding site in IL‐1β promoter. The transcription start site is indicated as TSS. Potential FOXC1 (blue color) and p65 (orange color) binding sites in IL‐1β promoter were marked. All FOXC1‐binding sites were predicted by the JASPAR database. The p65‐binding site is well established. The right panel shows IL‐1β promoter luciferase reporter assay results in MDA‐MB‐231 cells (*n* = 3). (G–I) Recruitment of FOXC1, p65, and Pol II to the *IL‐1β* gene. 231‐Vctrl and 231‐Foxc1 cells were stimulated with 1 μg/mL LPS for 4 h and ChIP assays were performed. The binding of (G) FOXC1, (H) p65 binding fold change, and (I) RNA Pol II to different regulatory regions of the *IL‐1β* gene was measured by qRT‐PCR (*n* = 3). Data represent mean ± SD of three independent experiments. Statistical significance: *p < 0.05; **p < 0.01; ***p < 0.001; ****p < 0.0001.

LPS‐mediated NF‐κB activation is known to promote IL‐1β transcription.^8^ We thus set out to explore whether FOXC1 is involved in this process. While FOXC1 overexpression did not alter the baseline expression of IL‐1β, it significantly inhibited the NF‐κB p65 overexpression‐induced increase in both IL‐1β RNA and protein levels (Figures 1D and E) as well as expression of Cox‐2 and ICAM1 (Figure 1E). We observed similar results in MDA‐MB‐436 and HCC1806 BLBC cells (Figure S4), indicating that FOXC1 directly affects the NF‐κB regulation of IL‐1β. It is noted that there is a difference in the trend of pro‐IL‐1β level changes presented in Figures 1C and 1E. This may be explained by transfection of p65 and FOXC1 in cells, resulting in a strong hinderance of IL‐1β expression by FOXC1.

FOXC1 is known to bind the nine‐base‐pair core sequence GTAAATAAA.^9^ and we identified two highly conserved FOXC1‐binding sites in the *IL‐1β* promoter region (Figure 1F), flanking a well‐established p65‐binding site.^8^ We then cloned the *IL‐1β* promoter fragment containing the predicted FOXC1‐binding sites and the p65‐binding site into the pGL4 luciferase reporter construct and performed luciferase assays (see Supplementary Methods). FOXC1 overexpression alone did not change the IL‐1β promoter luciferase activity. In contrast, p65 overexpression significantly increased the luciferase activity, confirming that p65 activates IL‐1β transcription (Figure 1F). Notably, this p65 effect was substantially attenuated by FOXC1 co‐expression. We further tested FOXC1 binding to the two sites using chromatin immunoprecipitation (ChIP) assays followed by qPCR analysis of the immunoprecipitated DNA in 231‐Foxc1 and 231‐Vctrl cells. Overexpression of FOXC1 significantly enhanced the recruitment of FOXC1 to binding site 1 (FS1) and binding site 2 (FS2) compared with the vector control (Figure 1G). Additionally, we also performed an NF‐κB luciferase reporter assay. As expected, FOXC1 overexpression increased NF‐κB‐driven luciferase activity in MDA‐MB‐231 (Figure S5). This is probably due to FOXC1‐mediated increase of p65 protein stability as demonstrated in previous studies.^4^ We then postulated that direct interactions between FOXC1 and the IL‐1β promoter may interfere with p65 access to its binding site, resulting in the suppression of IL‐1β induction. To test this hypothesis, we used LPS to activate NF‐κB. As demonstrated by p65 ChIP assays, overexpression of FOXC1 significantly reduced p65 binding to the *IL‐1β* promoter in response to LPS stimulation (Figure 1H). As RNA Polymerase II (Pol II) drives rapid gene transcription, we then investigated the effect of FOXC1 on Pol II binding to the two well‐documented sites in the IL‐1β promoter region.^10^ Pol II ChIP assays showed LPS increased Pol II recruitment to the *IL‐1β* gene, but this increase was diminished by FOXC1 overexpression (Figure 1I). To examine the effect of FOXC1 overexpression on breast cancer cell migration and invasion induced by LPS, transwell assays were conducted. We found that LPS significantly increased cell migration and invasion in 231‐Vctrl cells, while this LPS effect was attenuated by FOXC1 overexpression (Figures S6A and B).

In conclusion, our findings suggest anti‐inflammatory properties of FOXC1 in cancer cells and molecular mechanisms for FOXC1 as a novel regulator of IL‐1β and inflammation. FOXC1 is known to play an important role in various cancer types. The molecular mechanisms we uncovered can potentially apply to other cancer types as well. Together these findings reveal a new molecular connection between FOXC1 and tumor immunosuppression, presenting another conceptual framework for better understanding and exploiting of patient‐specific responses to cancer immunotherapy.

## AUTHOR CONTRIBUTIONS

Y. L., A. G., and X. C. conceived and designed the experiments. Y. L. performed experiments, analyzed data, and prepared the original draft of the manuscript. X. C. supervised the research, wrote, revised, and edited the manuscript. S. C. provided assistance with the experiments. S. C. and A. G. revised the manuscript. M. T. contributed to the analysis of TCGA data. All authors have read and approved the final manuscript.

## CONFLICT OF INTEREST STATEMENT

The authors declare that they have no conflict of interests.

## FUNDING INFORMATION

U.S. Department of Defense W81XWH‐18‐1‐0067; The National Institutes of Health 2R01CA151610, R21CA280458; Linda and Jim Lippman Fund.

## ETHICS STATEMENT

Not applicable.

## CONSENT FOR PUBLICATION

Not applicable.

## Supporting information

Supporting Information

## Data Availability

The data required to reproduce these findings are available from the corresponding author upon reasonable request.
