# Supplementary material for: FOXC1 restrains NF‐κB‐mediated interleukin‐1β transcription in breast cancer
Source: MedComm (2020). 2023 Dec 16;4(6):e440. doi: 10.1002/mco2.440 (PMC10725083; doi:10.1002/mco2.440)
Supplement: Supplementary file 1 — Supporting Information [file MCO2-4-e440-s001.docx]

**Title:** FOXC1 Restrains NF-κB mediated Interleukin-1β Transcription in Breast Cancer

**Running title**: Regulation of IL-1β by FOXC1

**Authors:** Yan Liu^1^, Shuang Chen^2^, Mao Tian^3^, Giuliano Armando^1^, Xiaojiang Cui^1, *^

**^1^** Department of Surgery, **^2^** Department of Biomedical Sciences, Cedars-Sinai Medical Center, Los Angeles, CA 90048, USA.

**^3^** Jonsson Comprehensive Cancer Center, the University of California, Los Angeles, CA 90024, USA

**Corresponding author:** Xiaojiang Cui, 8700 Beverly Blvd, Davis Bldg 2065, Los Angeles, CA 90048, USA. E-mail: Xiaojiang.cui@cshs.org

**Keywords:** FOXC1, IL-1β, Inflammation, NF-κB, Basal-like breast cancer

**Supplementary Materials and Methods**

**Cell culture and stimulation**

Human breast cancer cell line MDA-MB-231, MDA-MB-436 and HCC1806 were purchased from American Type Culture Collection (ATCC) and maintained according to ATCC instructions. The control and FOXC1-overexpressing MDA-MB-231 cells were established as previously described ^1^. Lipopolysaccharide (LPS, #L4391-1MG, Sigma Aldrich) was used as an inducer of immune response in MDA-MB-231 cells.

**Quantitative real-time RT-PCR**

Total RNA was extracted using the RNeasy Mini Kit (Qiagen) and reversely transcribed into single-stranded cDNAs using the iScript™ cDNA Synthesis Kit (Bio-Rad). Real-time PCR was performed using CFX96 Real-Time System (Bio-Rad). Primers were listed in the supplementary Table S1.

**Western blotting**

Proteins were extracted using lab made lysis buffer and protein concentration was determined by BCA Protein Assay Kit (Thermo Fisher). Proteins (40µg) were separated on 10% gradient gels and transferred onto NC membranes using wet transfer system (Bio-Rad). Membranes were blocked in Odyssey blocking buffer (LI-COR) and incubated with primary antibodies overnight at 4°C. The primary antibodies were used as follows: IL1B (1:2000, AF-201-NA, R&D Systems), Phospho-NF-κB p65 (Ser536) (1:1000, #3033, Cell Signaling), and ACTIN (1:1000, #SC-1616, Santa Cruz). The secondary antibodies were used as follows: IRDye 800CW secondary antibodies Goat anti-Mouse IgG (#926-32210, Li-Cor), Goat anti-Rabbit IgG (#926-32211, Li-Cor), Donkey anti-Goat IgG (#926-32214, Li-Cor). The membranes were scanned using the LI-COR imaging system.

**Luciferase reporter assay**

IL1β promoter fragments (-1455 to +63 bp) containing FOXC1-binding sites were cloned into luciferase vector pGL4 (Promega). The primers were listed in the supplementary Table S1. Cells seeded in 24-well plates were transfected with 200 ng luciferase plasmids and 200 ng pCMV6-entry (OriGene) or pCMV6-entry-FOXC1 (OriGene) expression plasmids using Lipofectamine 3000 (Invitrogen). Following treatment, firefly luciferase and Renilla luciferase activity were determined using the dual-luciferase assay kit (Promega). Luciferase activity was measured using a luminometer (GloMax-Multi Detection System, Promega).

**Chromatin Immunoprecipitation Assay**

5×10^6^ cells were collected, and ChIP assays were performed using the ChIP-IT@ Express Chromatin Immunoprecipitation Kit (#53008, Active Motif) according to the manufacturer’s instructions. Anti-FOXC1 antibody (ab227977, Abcam) -immunoprecipitated DNA was analyzed by real-time PCR. The primers were listed in the supplementary Table S1.

**Tumor cell migration and invasion assay**

2×10^4^ cells were re-suspended in 200µl 2% FBS medium and seeded into the upper compartment of a transwell chamber (Corning) or Matrigel invasion chamber (BD Biosciences) for migration or invasion assay, respectively. The lower compartment of the chamber was filled with 750 µl complete medium (DMEM supplemented with 10% FBS). After 14 hours (migration) or 24 hours (invasion) of incubation, cells remaining in the upper compartment were removed with a cotton swab, and the migrated or invaded cells were stained by using 0.5% crystal violet. Pictures were taken (EVOS™ FL Auto Imaging System, ThermoFisher) and the cells were counted using ImageJ software.

**Statistics**

Statistical analyses were performed using GraphPad Prism 6 Software. Statistical comparisons were determined via independent student’s t-test. Linear regression analyses and Pearson correlation coefficients were conducted for calculating correlations. Values were represented as mean ± SD of at least three independent experiments. The results were considered statistically significant at a *P-value* < 0.05 (*), < 0.01 (**) or < 0.001 (***).


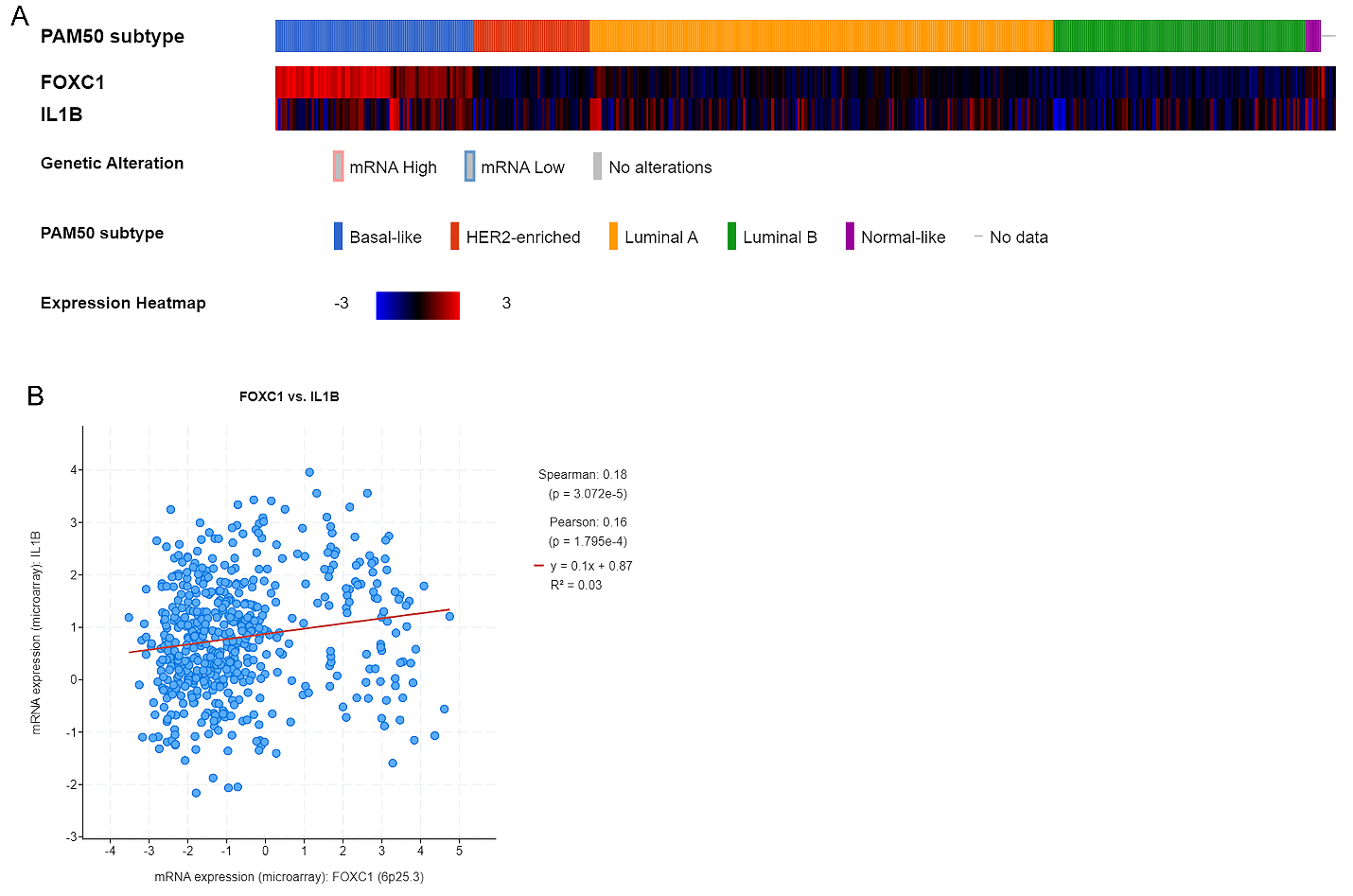
**Supplementary Figure S1.** **Differential expression of FOXC1 and IL-1β in human breast cancer subtypes** (A) Heatmap of FOXC1 and IL-1β mRNA expression in different breast cancer subtypes in the TCGA dataset ^2^. (B) Both Spearman and Pearson correlation analysis were conducted to examine the relationship between the mRNA levels of IL-1β and FOXC1 in TCGA dataset using cBioPortal. (n = 526, around 11% samples have a higher FOXC1 expression, around 4% samples have a higher IL-1β expression.). Linear regression analysis was performed to further investigate the relationship between these two genes.





**Supplementary Figure S2**. **LPS induces NF-κB activity in breast cancer cells**. MDA-MB-231 cells were transiently transfected with the NF-κB-luc construct. After 48 hrs of culture, the cells were stimulated with LPS for 4 hrs. The reporter activities were then measured using luciferase assays, and the results indicated a significant increase in NF-κB activity in response to LPS. Bars represent the mean ± SD (n = 3). Statistical significance: *** *P* < 0.0001.


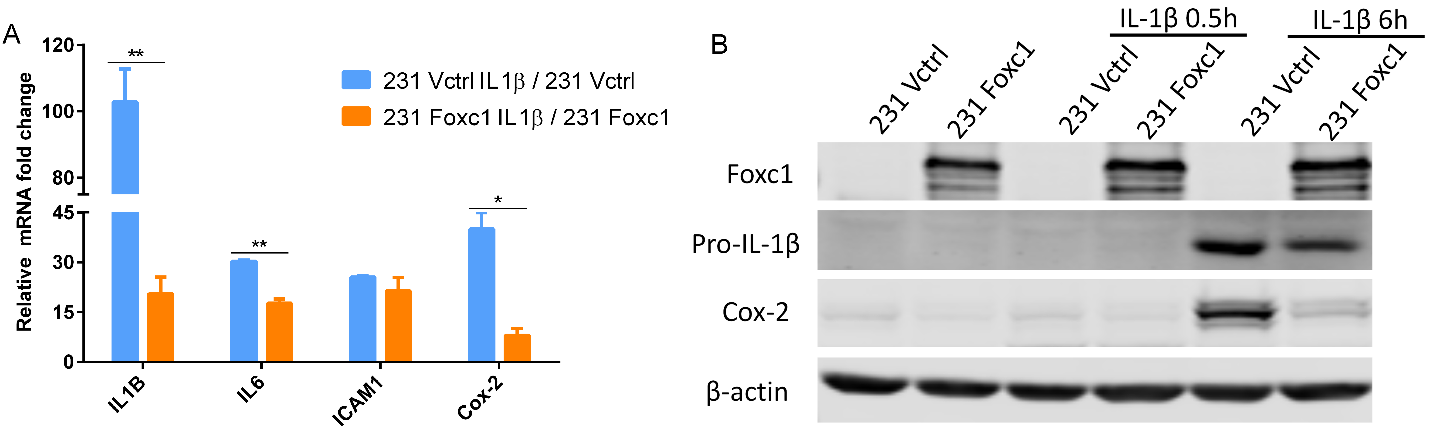


**Supplementary Figure S3. IL-1β triggers the inflammatory response in 231-Vctrl and 231-Foxc1 cells**. (A) FOXC1 inhibited inflammatory IL-1β, IL6, and Cox-2, but not ICAM1, mRNA expression in IL-1β (5 ng/mL)-stimulated MDA-MB-231 cells. mRNA levels were determined by Real-time PCR. (B) IL-1β (5 ng/mL) treatment in 231-Vctrl and 231-Foxc1 cells for 0.5 and 6 hours, cell lysates were subjected to western blotting analysis of FOXC1, pro-IL-1β and Cox-2 proteins. Bars represent the mean ± SD (n = 3). Statistical significance: **P* < 0.05; ***P* < 0.01.


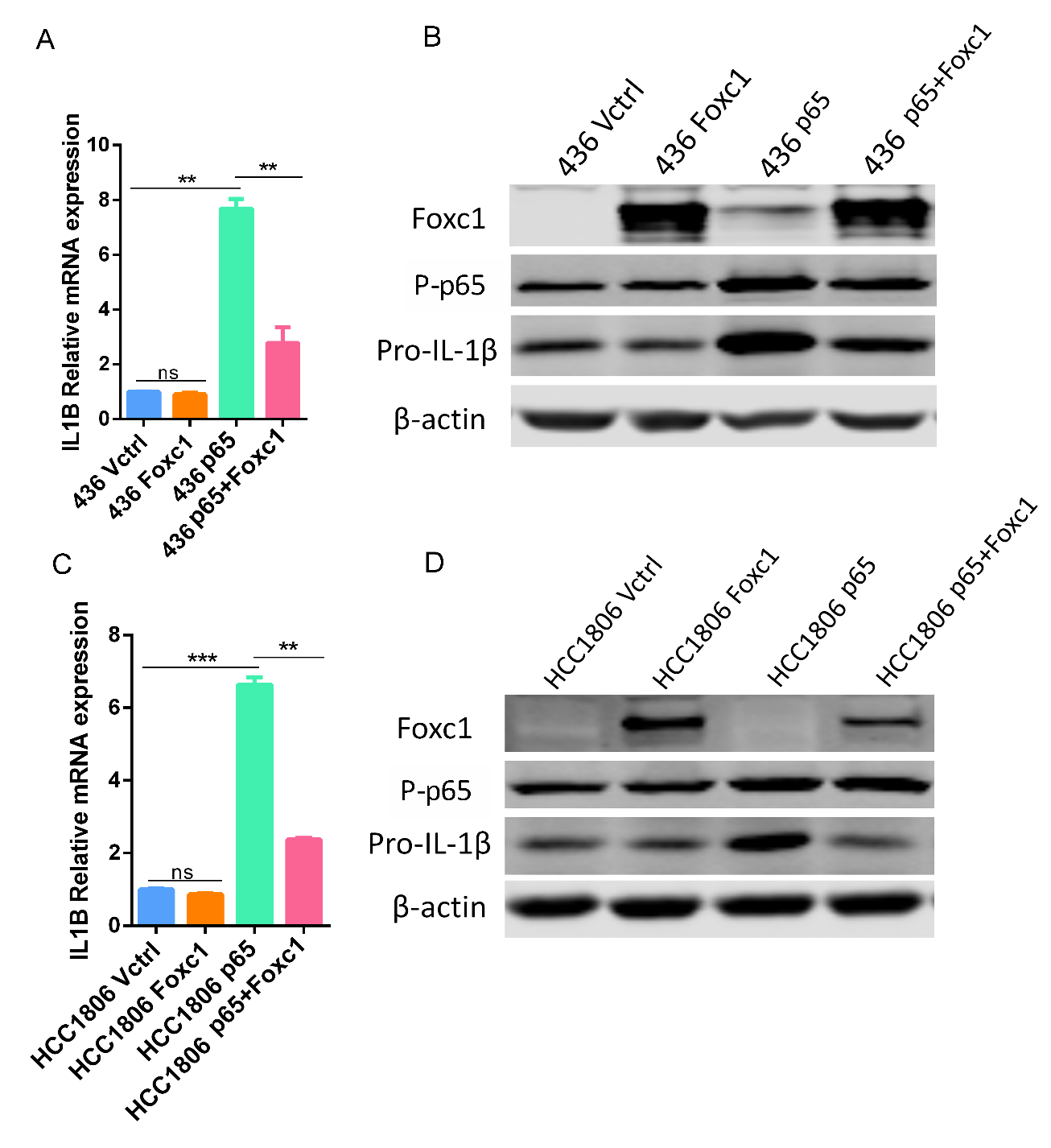


**Supplementary Figure S4. FOXC1 suppress NF-κB-induced regulation of the IL-1β in MDA-MB-436 and HCC1806 breast cancer cells.** MDA-MB-436 and HCC1806 transfected with PLNCX2-control (empty vector), PLNCX2-FOXC1 (FOXC1 expression vector), PCMV6-p65 (p65 expression vector) and co-transfected PLNCX2-FOXC1 and PCMV6-p65 vectors. qRT-PCR analysis of IL-1β mRNA expression in MDA-MB-436 and HCC1806 (A, C); Western blotting analysis of FOXC1, P-p65, pro-IL-1β and β-actin protein expression in MDA-MB-436 and HCC1806 (B, D). Bars represent the mean ± SD (n = 3). Statistical significance: ***P* < 0.01; ****P* < 0.0001.





**Supplementary Figure S5**. **FOXC1 induces NF-κB activity in breast cancer cells**. MDA-MB-231 cells were transiently transfected with NF-κB-luc with PLNCX2-control (empty vector) or PLNCX2-FOXC1 (FOXC1 expression vector). NF-κB activity was assessed by luciferase assays. Bars represent the mean ± SD (n = 3). Statistical significance: **P* < 0.05.


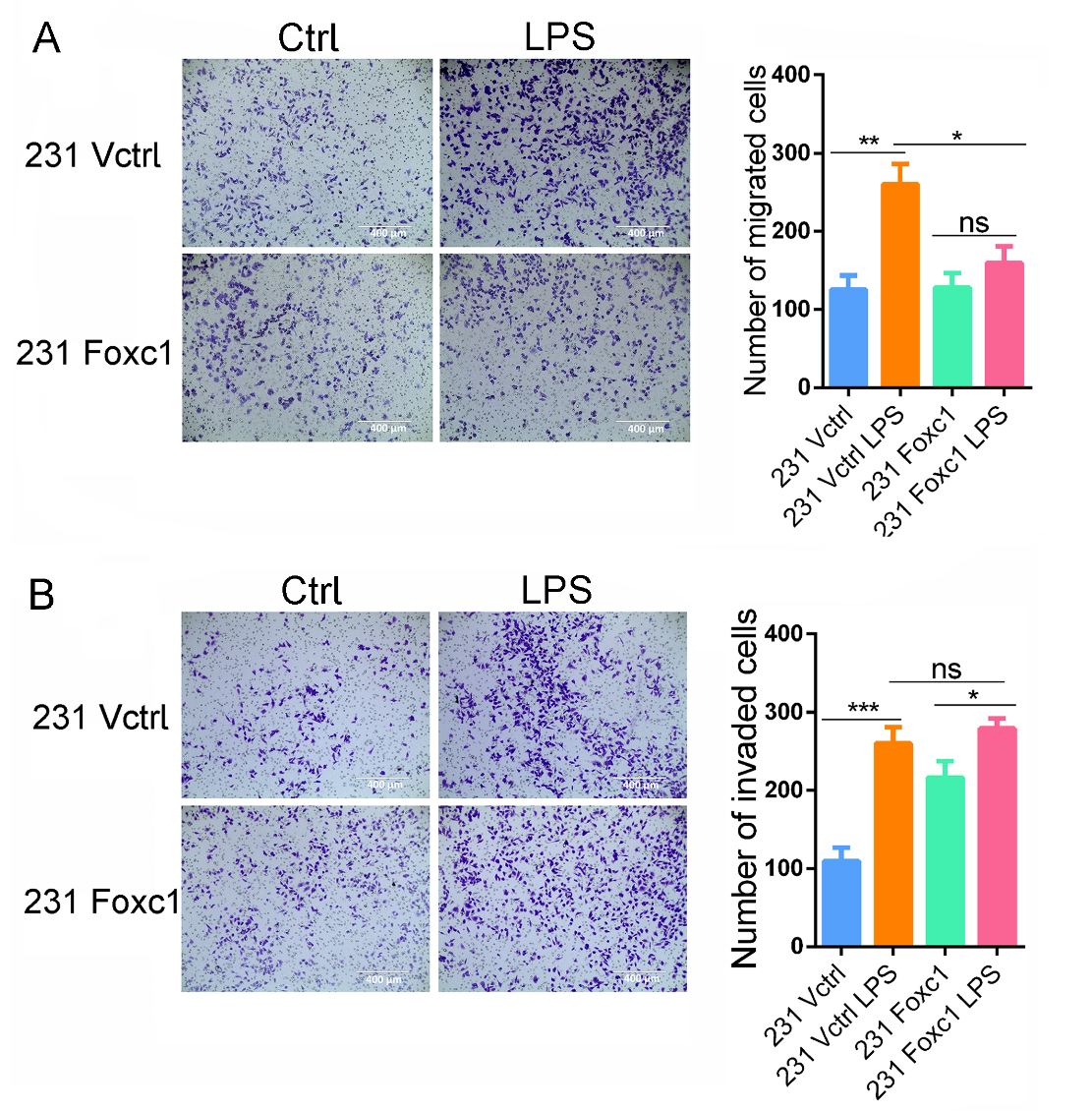


**Supplementary Figure S6**. Representative images showing migrated (A) and invaded (B) 231-Vctrl and 231-Foxc1 cells under normal or LPS conditions using transwell assays, which were quantified. Migrated cells were stained after 14 hrs of incubation and invaded cells were stained after 24 hrs of incubation. Scale bar, 400 µm. Bars represent the mean ± SD (n = 6). Statistical significance: **P* < 0.05; ***P* < 0.01; ****P*< 0.001.

**References**

1. Ray PS, Wang J, Qu Y, et al. FOXC1 is a Potential Prognostic Biomarker with Functional Significance in Basal-like Breast Cancer. *Cancer Res*. 2010;70(10):3870-3876.

2. Koboldt DC, Fulton RS, McLellan MD, et al. Comprehensive molecular portraits of human breast tumours. *Nature*. 2012/10/01 2012;490(7418):61-70. doi:10.1038/nature11412

**Supplementary Table S1**

| **RT-PCR Primers** |  |  |
| --- | --- | --- |
|  | **Forward (5’-3’)** | **Reverse (5’-3’)** |
| **FOXC1** | GGCAAAGAATTGATCCGGTA | TGGATGGCCATGGTGATGAGC |
| **IL1B** | CCACAGACCTTCCAGGAGAATG | GTGCAGTTCAGTGATCGTACAGG |
| **IL6** | AGACAGCCACTCACCTCTTCAG | TTCTGCCAGTGCCTCTTTGCTG |
| **ICAM1** | AGCGGCTGACGTGTGCAGTAAT | TCTGAGACCTCTGGCTTCGTCA |
| **COX-2** | CGGTGAAACTCTGGCTAGACAG | GCAAACCGTAGATGCTCAGGGA |
|  |  |  |
| **IL-1β promoter clone primers** | |  |
| **IL1B promoter C1** | CGGGGTACCCGATACCTGGCACATACTAA | CTAGCTAGCTGAAGATTGGCTGAAGAGAA |
|  |  |  |
| **ChIP Primers** |  |  |
| **FOXC1 Binding site 1 (FS1)** | CATCAACTGCACAACGATT | ATTCTCTGGTTCATGGAAGG |
| **FOXC1 Binding site 2 (FS2)** | TGTGTGTCTTCCACTTTGT | TCGTTGTGCAGTTGATGT |
| **p65 Binding site (NF-κB)** | ATCTGTGTGTCTTCCACTTTGTC | CAATCGTTGTGCAGTTGATGTC |
| **Pol II (+36)** | AAACCTCTTCGAGGCACAAG | GAGCAATGAAGATTGGCTGA |
| **Pol II (+136)** | ACAACTAGGTGCTAAGGGAGTC | AGGAGAGGGAGAGACAGAGAAAGA |
| **GAPDH (ChIP)** | TACTAGCGGTTTTACGGGCG | TCGAACAGGAGGAGCAGAGAGCGA |
